# The Fabrication of Ultrahigh-Strength Steel with a Nanolath Structure via Quenching–Partitioning–Tempering

**DOI:** 10.3390/ma17051161

**Published:** 2024-03-01

**Authors:** Wenting Xu, Li Xie, Xiaoying Liu, Jiangnan Wang, Yuxuan Xu, Mingtao He, Kejun Hu, Chang Liu, Wei Yu

**Affiliations:** 1 School of Materials Engineering, Jiangsu University of Technology, Changzhou 213001, China; xuwenting0408@163.com (W.X.); 15195800828@163.com (X.L.); 18900633907@163.com (J.W.); 15195489568@163.com (Y.X.); hemingtaotao@163.com (M.H.); kejun@jsut.edu.cn (K.H.); 2 Beijing Beiye Functional Materials Corporation, Beijing 100192, China; 17351595985@163.com; 3 Institute of Engineering Technology, University of Science and Technology Beijing, Beijing 100081, China

**Keywords:** ultrahigh-strength steel, quenching–partitioning–tempering (Q-P-T), microstructure, mechanical property

## Abstract

A novel low-alloy ultrahigh-strength steel featuring excellent mechanical properties and comprising a nanolath structure was fabricated in this work using a quenching–partitioning–tempering (Q-P-T) process. The Q-P-T process comprised direct quenching and an isothermal bainitic transformation for partitioning after thermo-mechanical control processing (online Q&P) and offline tempering (reheating and tempering). The ultrafine nanolath martensite/bainite mixed structure, combined with residual austenite in the form of a thin film between the nanolaths, was formed, thereby conferring excellent mechanical properties to the steel structures. After the Q-P-T process, the yield and tensile strengths of the steels reached 1450 MPa and 1726 MPa, respectively. Furthermore, the Brinell hardness and elongation rate were 543 HB and 11.5%, respectively, with an average impact energy of 20 J at room temperature.

## 1. Introduction

The rapid development of economy and the intensification of energy crises have resulted in an increasing demand for ultrahigh-strength steels (referring to steels with a tensile strength of approximately 1500 MPa) [1,2,3,4,5]. Ultrahigh-strength steel is widely used in fields such as the power industry, building materials, metallurgy, coal mining, and agricultural machinery. Its applications include excavator buckets, coalmine scraper conveyors, crusher jaws, and various high-precision molds [6,7,8]. Especially, with the increasing demand for lightweight automobiles, the application of ultrahigh-strength steels in automobiles has attracted great attention, which can not only bring decreased energy consumption and reduced greenhouse gas emissions, but also improve the passive safety of automobiles. According to relevant data statistics, a reduction of 100 kg in a vehicle’s weight corresponds to a reduction of 0.4 L/100 km in fuel consumption and 10 g/100 km in CO_2_ emissions.

Widely used ultrahigh-strength steels, such as AerMet100 [9,10,11,12], AF1410 [13,14,15], and HY180 [16,17], contain large amounts of precious alloys. This leads to high raw material costs, difficult smelting, and complex rolling and heat-treatment processes. In addition, owing to the special working environment of ultrahigh-strength steels, the requirements for hardness and wear resistance are increasing [18,19]. For low-alloy ultrahigh-strength steels (4340, 4140, 300M, 30CrMnSiNi2A, 40CrNi2Si2MoVA, etc.), the content of precious alloy elements still remains high [20,21,22,23,24]. Meanwhile, conventional high-strength steels are hardened by grain refinement, precipitation, or solid solutions. However, ultrahigh-strength steels are hardened mainly through rapid phase transformations, although they often have microstructural parameters that differ from each other [25]. Their microstructures generally include martensite, bainite, and retained austenite.

In general, the heat treatment of ultrahigh-strength steels is complex and mainly involves offline quenching (reheating and quenching) and partitioning (offline Q&P). The Q&P process was first proposed by J. Speer et al. [26] and D. K. Matlock et al. [27] in 2003 to improve the mechanical properties of steels. Q&P steels mainly utilize a heat treatment to partially stabilize ɣ through the use of carbon partitioning combined with a considerable amount of Si to inhibit cementite precipitation, and their microstructures contain martensite, and potentially ferrite, along with significant amounts of retained austenite stabilized by high carbon contents [28,29]. Carbon-stabilized austenite is obtained via carbon transfer from martensite into austenite after a controlled amount of martensite is introduced by judicious selection of a so-called quench temperature, at which quenching below the martensite start temperature is interrupted [29]. This complex manufacturing technique was proposed on the basis that only carbon can diffuse sufficiently rapidly beyond unit cell dimensions below the intercritical annealing regime [30]. Ultimately, Q&P steels allow cold forming of automotive components, which is not possible with conventional high-strength steels of comparable strength levels [31]. In addition, Q&P steels’ approach to retaining austenite results in refined microstructures with elevated strengths [32] and improved elongations, compared to their quenched and tempered counterparts [33,34]. These steels are first either fully austenitized or intercritically annealed, then subjected to an intermediate quench temperature between the martensite start and martensite finish temperatures and held at such temperature (for one-step Q&P) or above the Ms temperature (for two-step Q&P) [34,35].

Because there are no carbide-forming elements in Q&P steels (such as Nb, V, Ti, Mo, B, etc.), the advantage of precipitation strengthening and fine-grain strengthening is excluded [36]. However, a new type of composition, containing micro-alloying elements (such as Nb, V, Ti, Mo, B, etc.), has been designed to improve the strength and ductility on the basis of the Q&P process, namely the quenching–partitioning–tempering (Q-P-T) process [37,38,39]. The Q-P-T process specifically includes a quenching procedure from the austenization to the temperature between the martensite start and the martensite finishing point and an isothermal partitioning/tempering procedure, in which carbon diffuses from saturated martensite into adjacent untransformed austenite and stable carbides such as NbC are precipitated from the martensite matrix [26,40].

In this study, a novel ultrahigh-strength steel comprising a nanolath structure and exhibiting excellent mechanical properties was fabricated via the Q-P-T process containing direct quenching and isothermal bainitic transformation for partitioning after thermo-mechanical control processing (online Q&P) and offline tempering (reheating and tempering). Cheaper elements such as Mn and Si were used to increase the strength and wear resistance. Grain-refinement elements (Nb and V) were added to reduce the content of precious alloy elements, i.e., B and Ni. In addition, the C content was kept below 0.25%, which improved the weldability of the ultrahigh-strength steel and made it more versatile. Finally, a multi-phase mixed structure with nanolath martensite/bainite, plus residual austenite in the form of a thin film between the laths, was obtained, leading to excellent mechanical properties. The effects of the direct isothermal bainitic transformation time and the offline tempering temperature on the multi-phase mixed structure and the mechanical properties were investigated.

## 2. Materials and Methods

The material used in this study, for which the chemical composition is presented in Table 1, was obtained via smelting in a 25 kg vacuum induction furnace and forged into 80 mm × 80 mm × 80 mm billets.

**Figure 1:**
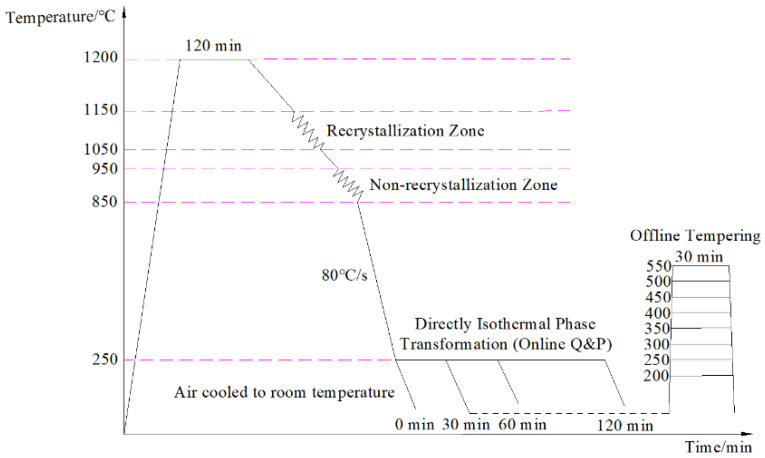
Schematic of hot-rolling and heat-treatment processes of the steels.

The billets were heated to 1200 °C for 120 min, hot-rolled to a thickness of 12 mm (with a total reduction rate of 85% through two stages of hot rolling using a D350 rolling mill), and air-cooled to room temperature. The first stage of hot rolling was performed in a dynamic recrystallization zone between 1050 °C and 1150 °C, and the total reduction was 52.5%. The second stage of hot rolling was performed in non-dynamic recrystallization zone between 850 °C and 950 °C, and the total reduction was 68.4%. After the two-stages of hot rolling, the steels were water-cooled to 250 °C for direct isothermal bainitic transformation (online Q&P), with high-density piping at a cooling rate of 80 °C/s. In addition, after direct isothermal bainitic transformation for 30 min at 250 °C for the online Q&P, the steels were offline-tempered at 200, 250, 300, 350, 400, 450, 500, and 550 °C for 30 min. The two stages of hot-rolling and heat-treatment processes are shown in Figure 1.

The tensile test was performed using a electric universal testing machine (ZLC, CMT 4105). The impact test was conducted at room temperature using standard Charpy V-notch impact specimens with a JB-30B impact tester. The impact fracture morphology and the microstructure were examined via scanning electron microscopy (SEM, LEO-1450). High-resolution transmission electron microscopy (TEM, JEM-2010) was performed to examine the microstructure and precipitated particles. The component of precipitated particles was analyzed with energy dispersive spectrometry (EDS). The residual austenite content in the steels was investigated using X-ray diffraction (XRD, D8 ADVANCE). The Brinell hardness was evaluated using a Brinell hardness tester (King, BLD-3000TS).

## 3. Results

### 3.1. Mechanical Properties

**Figure 2:**
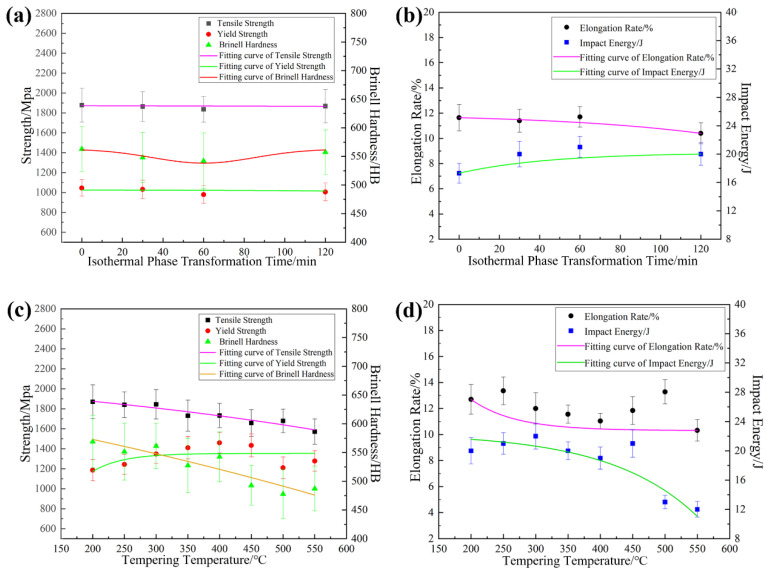
Effects of the processes on the mechanical properties, Brinell hardness, and impact energy at room temperature. The effects of the direct isothermal bainitic transformation time on (**a**) strength and Brinell hardness and (**b**) elongation rate and the impact energy. The effects of the tempering temperature on (**c**) strength and Brinell hardness and (**d**) elongation rate and the impact energy.

The effects of the direct isothermal bainitic transformation time and the offline tempering temperature on the mechanical properties, Brinell hardness, and impact energy at room temperature are shown in Figure 2.

After the two stages of hot rolling, the steels were water-cooled to 250 °C for direct isothermal bainitic transformation, with high-density piping at a cooling rate of 80 °C/s for the online Q&P process. During this period, with the increase in the isothermal bainitic transformation time, the tensile and yield strength were maintained at approximately 1800 and 1000 MPa, respectively, as shown in Figure 2a. In other words, the isothermal bainitic transformation time had a minor impact on strength. The Brinell hardness first decreased and then increased, as shown in Figure 2a. The variations in the elongation rate and impact energy at room temperature with respect to the isothermal bainitic transformation time are shown in Figure 2b. With the increase in the isothermal bainitic transformation time, the impact energy was found to increase, but the elongation rate showed a slight decline.

As shown in Figure 2c,d, with the increase in offline tempering temperature, the tensile strength and Brinell hardness decreased significantly, the yield strength increased gradually, the elongation rate maintained a smooth downward trend, and the impact toughness at room temperature tended to decrease. Moreover, when the offline tempering temperature was below 500 °C, the changes in Brinell hardness, elongation rate, and impact energy at room temperature were insignificant, yet the yield strength increased significantly. In contrast, when the tempering temperature exceeded 500 °C, the yield strength, Brinell hardness, and impact energy at room temperature decreased significantly, indicating that the steels had entered the tempering embrittlement zone.

### 3.2. Microstructures

#### 3.2.1. Effect of the Direct Isothermal Bainitic Transformation Time on the Microstructure

**Figure 3:**
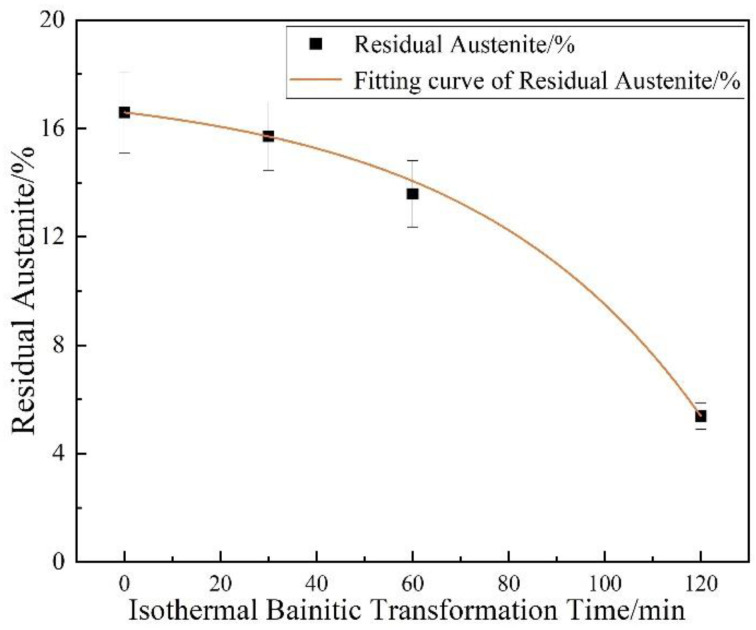
Effect of the isothermal bainitic transformation time on the residual austenite content.

The relationship between the residual austenite content and the isothermal bainitic transformation time is presented in Figure 3. With the increase in the isothermal bainitic transformation time, the residual austenite content showed a rapidly declining trend. As the isothermal bainitic transformation time increased to 120 min, the amount of residual austenite in the steels decreased by nearly two-thirds compared with the initial hot-rolling state (from 16.59% to 5.38%), as shown in Figure 3.

**Figure 4:**
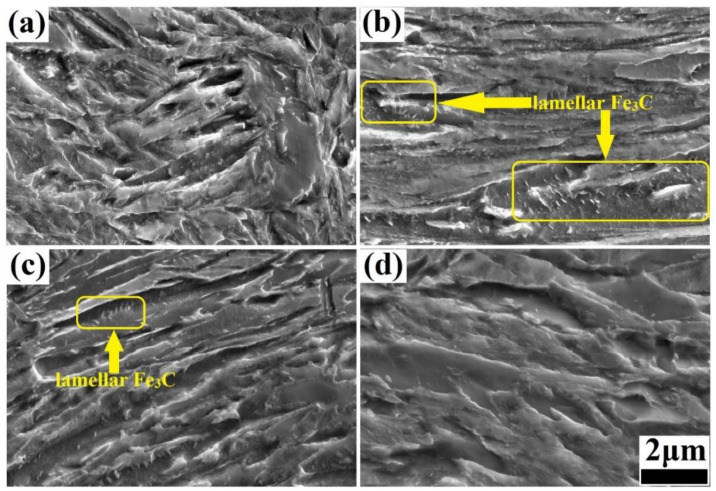
Microstructure obtained via SEM after different isothermal bainitic transformation times: (**a**): 0 min (the hot-rolling state); (**b**): 30 min; (**c**): 60 min; (**d**): 120 min.

The microstructure of the steels was obtained via SEM under different isothermal bainitic transformation times, including 0 (the hot-rolling state), 30, 60, and 120 min, as shown in Figure 4. With the extension of the isothermal bainitic transformation time, the microstructure of bainite/martensite nanolaths transformed gradually from an irregular stack structure to a homogeneous lamination structure, as shown in Figure 4a,c. The nanolath bundle became more obvious, and the brightness of the nanolaths decreased gradually, indicating that the C content of the nanolaths had decreased significantly. When the isothermal bainitic transformation time reached 120 min, the microstructure had clearly undergone self-tempering, as shown in Figure 4d. In addition, a large amount of lamellar Fe_3_C was produced on the nanolaths through the diffusion of the C atoms during the early stages of online Q&P, as shown by the rectangular areas in Figure 4b,c. As the direct isothermal bainitic transformation time increased, the C atoms further diffused and migrated, resulting in the dissolution of a large amount of lamellar Fe_3_C on the nanolaths. When the isothermal bainitic transformation time reached 120 min, no lamellar Fe_3_C was observed in the matrix, as shown in Figure 4d.

#### 3.2.2. Effect of the Offline Tempering Temperature on the Microstructure

**Figure 5:**
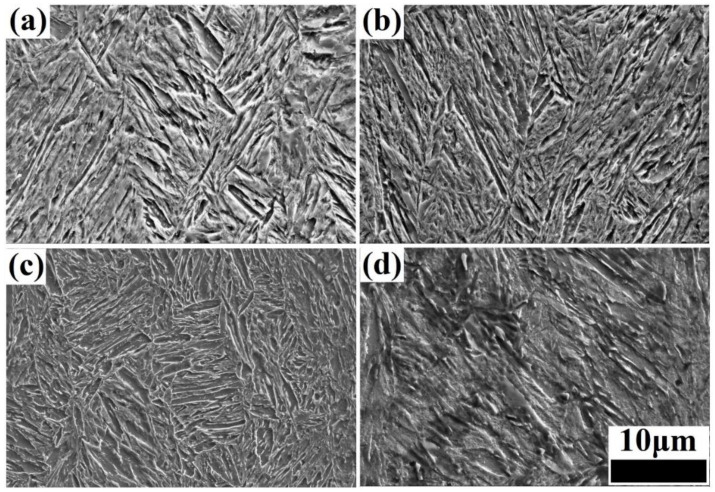
Microstructures obtained via SEM after direct isothermal bainitic transformation for 30 min at 250 °C and offline tempering at (**a**) 200 °C, (**b**) 300 °C, (**c**) 400 °C, and (**d**) 500 °C.

The microstructure obtained via SEM after offline tempering is presented in Figure 5. In comparison to the microstructures obtained before offline tempering, as shown in Figure 4, most of the nanolath bundles running through the grain had disappeared after offline tempering, and small nanolath bundles with different orientations were formed inside the grain at low tempering temperatures (200–300 °C), as shown in Figure 5a,b. Additionally, as the offline tempering temperature increased, the number of nanolath bundles increased, refining the microstructure, and improving its uniformity. When the offline tempering temperature continued to increase (350–450 °C), the nanolath width further increased, and the residual austenite, in the form of a thin film between the nanolaths, became thinner, as shown in Figure 5c. The nanolaths clearly tended to be uniform. Moreover, the amount of Fe_3_C, in the form of a thin film between the nanolaths, significantly decreased, although it did not disappear completely. In addition, a few second-phase particles precipitated on the matrix. This indicated that part of the residual austenite was dissolved during the offline tempering, forming carbide and lamellar bainite. When the tempering temperature exceeded 500 °C, the nanolath structure became more uniform, as shown in Figure 5d. However, the nanolath width increased significantly, reaching approximately 1 μm.

#### 3.2.3. TEM Microstructure Observation

**Figure 6:**
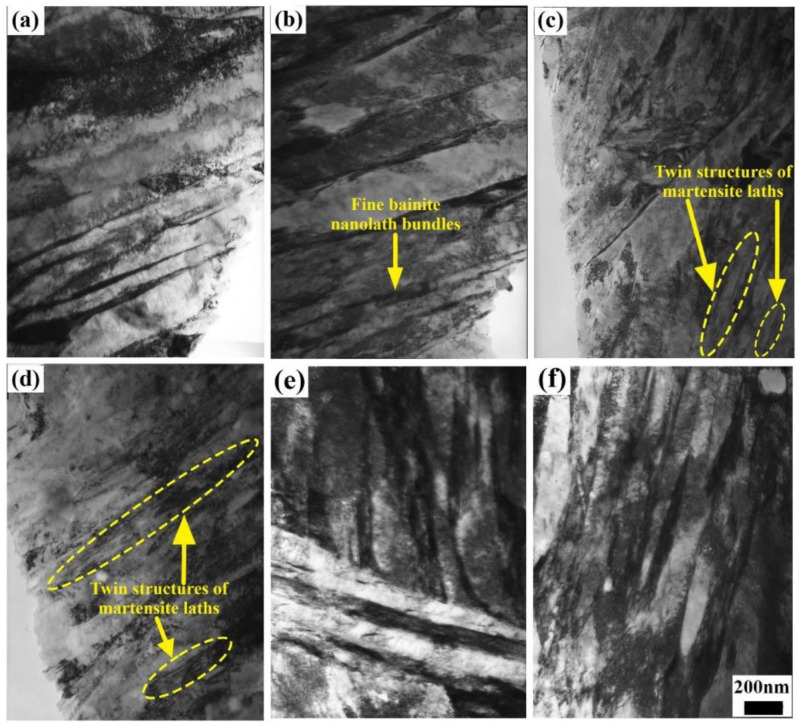
TEM morphologies of the microstructure in steels under different heat-treatment processes: isothermal bainitic transformation for 30 min (**a**,**c**) and 60 min (**b**,**d**) at 250 °C; isothermal bainitic transformation for 30 min at 250 °C and offline tempering for 30 min at (**e**) 300 °C and (**f**) 400 °C.

The TEM morphology images of the steels under different heat-treatment processes are shown in Figure 6. All of the microstructures of the steels after online Q&P and Q-P-T were nanolath martensite/bainite, plus residual austenite in the form of a thin film between the nanolaths. After the online Q&P process involving direct isothermal bainitic transformation for 30 and 60 min at 250 °C, the martensite/bainite laths, whose width was approximately 200 nm, were clearly visible, as shown in Figure 6a,b. Meanwhile, the twinning lamellar structure of martensite laths was obtained by martensite phase transformation, as shown by the dotted zones in Figure 6c,d. The residual austenite underwent bainite transformation during the subsequent online partitioning process, forming numerous fine bainite nanolath bundles (<100 nm), as indicated by the arrow in Figure 6b. Furthermore, numerous bainite nanolaths with different orientations and widths of <100 nm were formed in the grains after Q-P-T, as shown in Figure 6e,f.

#### 3.2.4. Second-Phase Particle Precipitation

**Figure 7:**
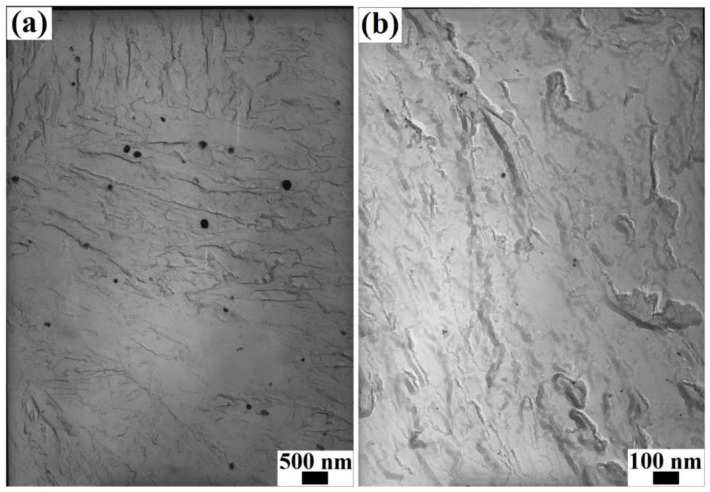
TEM morphologies of the precipitated particles in the steels obtained under different processing conditions: (**a**): isothermal bainitic transformation for 30 min at 250 °C; (**b**): isothermal bainitic transformation for 30 min at 250 °C and offline tempering for 30 min at 400 °C.

**Figure 8:**
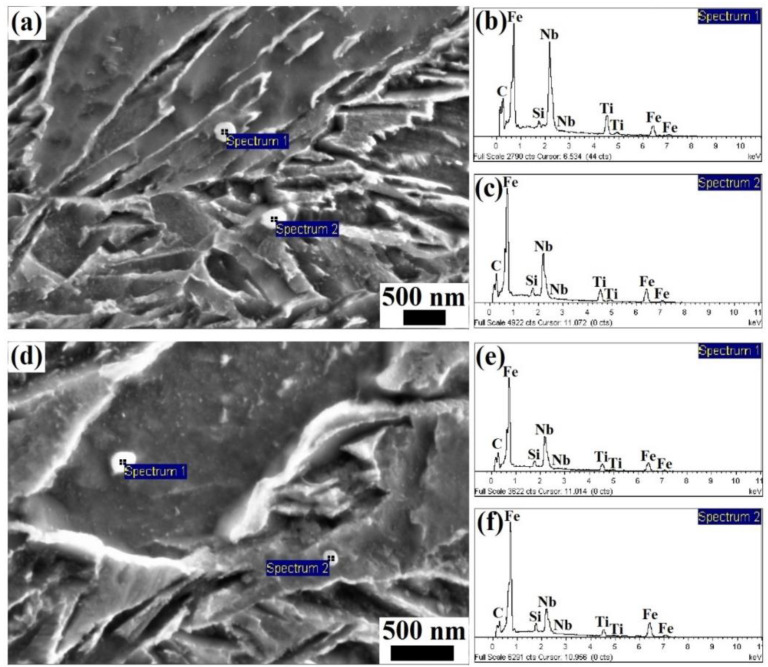
EDS spectra of the precipitated particles in steels under different processes: (**a**–**c**): isothermal bainitic transformation for 30 min at 250 °C; (**d**–**f**): isothermal bainitic transformation for 30 min at 250 °C and offline tempering for 30 min at 400 °C.

The TEM morphologies (obtained via the extraction replica technique) and the EDS spectra of the precipitated particles in the steels obtained under different processing conditions are given in Figure 7 and Figure 8, respectively. The size of the precipitated particles was determined to be approx. 60–100 nm in the steels after online Q&P, as shown in Figure 7a. The EDS spectra indicated that the precipitated particles were NbC, as shown in Figure 8a,b, which were formed during the direct isothermal bainitic transformation after hot rolling for online partitioning. The precipitation morphologies and EDS spectra of the precipitated particles in the steels after the Q-P-T process are shown in Figure 7b and Figure 8d,f, respectively. Numerous small spherical and approximately spherical second-phase particles with sizes of approx. 10–20 nm were precipitated on the substrate, as shown in Figure 7b.

### 3.3. Fracture Morphology

**Figure 9:**
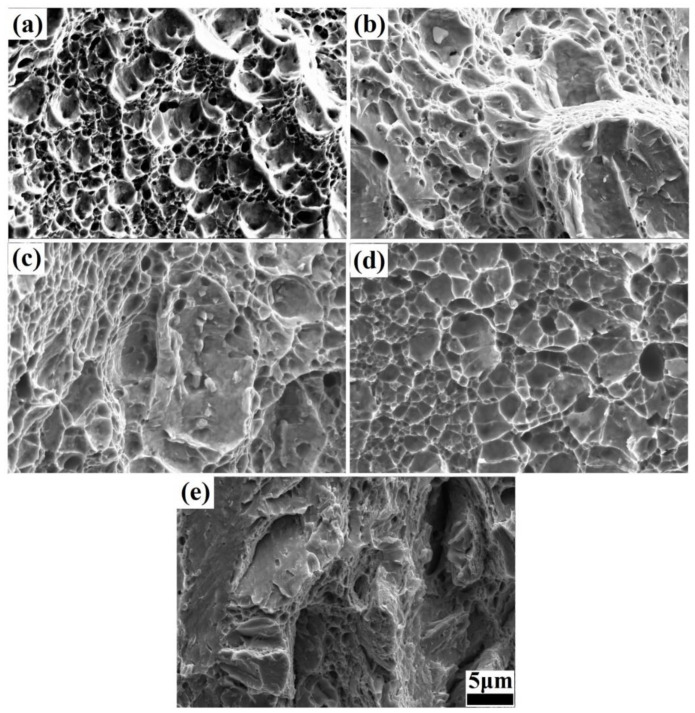
Fracture morphologies of the steels under different processing conditions: (**a**): hot-rolling state; (**b**): isothermal bainitic transformation for 30 min at 250 °C; isothermal bainitic transformation for 30 min at 250 °C and offline tempering for 30 min at (**c**) 300 °C, (**d**) 400 °C, and (**e**) 550 °C.

The fracture morphologies under different isothermal bainitic transformation times and offline tempering temperatures were examined via SEM, containing the hot-rolling state, as shown in Figure 9. Numerous dimples were exhibited in the hot-rolling state, as shown in Figure 9a. However, the dimples were relatively shallow, with a relatively large size. Moreover, there were tearing marks on the edges, resulting in low impact toughness. The fracture morphology after the 30 min isothermal bainitic transformation mainly consisted of quasi-cleavage surfaces and dimples, as shown in Figure 9b. Additionally, the dimple area was significantly reduced, accounting for approximately 40% of the fracture surface. However, the dimples were deeper and had smaller diameters than those formed in the hot-rolling state, indicating that more impact energy had been consumed. After the Q-P-T process involving isothermal bainitic transformation for 30 min at 250 °C and offline tempering for 30 min at 300 °C, the fracture morphology still consisted of quasi-cleavage surfaces and dimples, as shown in Figure 9c. However, the dimple area was reduced, accounting for approximately 25% of the fracture surface. The cracks formed at and propagated along the grain boundary, resulted in an intergranular fracture, as shown in Figure 9d. After the Q-P-T process involving direct isothermal bainitic transformation for 30 min at 250 °C and offline tempering for 30 min at 550 °C, the fracture surfaces were relatively uneven and smooth (the cleavage pattern), indicating a typical cleavage fracture, as shown in Figure 9e.

## 4. Discussion

The various trends observed in the mechanical properties, Brinell hardness, and impact energy at room temperature in the steels (shown in Figure 2) could be attributed to the combined effects of the transformation of residual austenite into bainite, the diffusion of C atoms, the formation and dissolution of Fe_3_C and martensite twinning, the precipitation strengthening of the particles, and so on. For example, during the direct isothermal bainitic transformation after the hot rolling for online partitioning, the martensite/bainite transformation started simultaneously. The transformation of martensite was completed instantaneously, whereas the bainite transformation occurred at a slower rate. Therefore, the bainite transformation was easily hindered by adjacent martensite that nucleated and grew earlier in the grains. Hence, the bainite grew independently and formed the nanolath substructures, refining the microstructure. As shown in Figure 6a–d, the nanoscale widths of the martensite/bainite laths and twinning lamellar structure of martensite laths were the main reason for the ultrahigh strength and high hardness demonstrated in Figure 2a. Meanwhile, during the direct isothermal bainitic transformation after the hot rolling for online partitioning, the C atoms diffused, resulting in the nucleation and growth of precipitated particles on the martensite/bainite nanolaths. Numerous particles with sizes of approx. 60~100 nm were precipitated onto the substrate, resulting in precipitation strengthening, as shown in Figure 7a. At the beginning of the isothermal phase transformation, C atom diffusion and the dissolution or disappearance of Fe_3_C played a dominant role. With the diffusion of C atoms on bainite/martensite nanolaths and the dissolution or disappearance of Fe_3_C particles, the Brinell hardness decreased. As the holding time increased, the amount of residual austenite that transformed into bainite increased, and the residual austenite played a dominant role. The relationship between the residual austenite content and the isothermal phase transformation time is presented in Figure 3. As the isothermal phase transformation time increased to 120 min, the amount of residual austenite in the steels decreased by nearly two-thirds compared with the initial hot-rolling state (from 16.59% to 5.38%). A large amount of soft-phase residual austenite was transformed into hard-phase bainite, increasing the hardness. In addition, in the later stage of phase transformation, large second-phase particles precipitated in the matrix, increasing the hardness. Thus, the tensile strength, yield strength, and Brinell hardness of the steels reached 1800 MPa, 1000 MPa, and 550 HB, 20 J, respectively, as shown in Figure 2a.

Because the lamellar Fe_3_C on and between the bainite/martensite nanolaths gradually dissolved as the isothermal phase transformation time increased, the elongation rate essentially remained unchanged during the initial phase transformation, and it decreased as the isothermal phase transformation time increased, as shown in Figure 2b. The elongation rate essentially remained unchanged in the early stage of phase transformation, which was due to the combined effect of three processes: the gradual dissolution of the lamellar Fe_3_C on and between the bainite/martensite nanolaths, the precipitation of second-phase particles, and the transformation of residual austenite into bainite. In the later stage of phase transformation, as the isothermal phase transformation time increased, the precipitation of the second-phase particles gradually dominated. Numerous second-phase particles precipitated at the boundary of the nanolaths, which pinned the dislocation and hindered the slip of the dislocation. This caused dislocation accumulation and stress concentration, forming microcracks and reducing the elongation rate. At the same time, the dissolution and disappearance of a large amount of the brittle Fe_3_C was crucial for the stability of the mechanical properties, resulting in a rapidly increasing trend for the impact energy at room temperature, as shown in Figure 2b. A large amount of lamellar Fe_3_C was produced on the nanolaths, caused by the diffusion of the C atoms during the early stages of the online Q&P. As the direct isothermal bainitic transformation time increased, the C atoms further diffused and migrated into the bainite/martensite nanolaths, resulting in the dissolution of lamellar Fe_3_C on the nanolaths.

The main changes that occurred during the offline tempering of the steels were as follows: dislocation tangle recovery, C atom diffusion, dissolution and disappearance of Fe_3_C, martensite nanolath recovery and polygonization, decomposition of twin martensite, residual austenite decomposition, nucleation and growth of carbide, and microstructure homogenization. Numerous bainite nanolaths with different orientations and widths of <100 nm were formed in the grains after the Q-P-T process, as shown in Figure 6e,f, which refined the microstructure and resulted in excellent mechanical properties. In general, a basic martensitic microstructure includes dislocations, grain boundaries, and the crystal lattice itself [41]. Because the martensite nanolath bundles tended to recover and polygonize during the offline tempering, the nanolath boundaries were gradually blurred, and the microstructure tended to be uniform. Moreover, the dislocation density was significantly reduced by static recovery, and the twinning lamellar structures of the martensite laths were rapidly decomposed. This manifested in significant reductions in the Brinell hardness and tensile strength, as shown in Figure 2c. Due to the comprehensive effect of precipitation strengthening and transformation-induced plasticity (caused by the residual austenite decomposing into bainite and carbide), the yield strength had a minor increase and the impact energy at room temperature was almost invariant before the tempering embrittlement zone (Figure 2c,d). In addition, the precipitation strengthening of the second-phase particles played a role in the increase in the yield strength at the beginning of the offline tempering, as shown in Figure 2c. The NbC mixed-metal precipitates were nano-size precipitates with a relatively homogeneous distribution in martensite/bainite nanolaths, as shown in Figure 7. The coherency of the particles depended on their size and shape, which was correlated with the tempering temperature [42,43,44]. With the increasing tempering temperature, the residual austenite, in the form of a thin film between the nanolaths, decomposed and formed carbides and second-phase particles. As the tempering temperature increased further, the second-phase particles continuously grew. Large carbides and second-phase particles that precipitated at the grain boundaries led to the emergence of the tempering brittle zone, as shown in Figure 2d.

It has been previously reported that the accumulation of dislocations close to the prior austenite grain boundaries, which form the intergranular crack path, are the preferential sites of accumulation of dislocation [41,45]. With an increase in the isothermal bainitic transformation time for online partitioning, the C atoms and lamellar Fe_3_C between the martensite/bainite nanolaths diffused and dissolved; thus, the fracture morphology after 30 min of isothermal bainitic transformation mainly consisted of quasi-cleavage surfaces and dimples, as shown in Figure 9b. On the quasi-cleavage surface, the direction of the main crack was unclear. The cracks originated within the grains, and their propagation gradually transitioned from cleavage steps to tearing ridges. Moreover, there were many short and curved tearing ridges inside the grains. These tearing ridges were caused by the lamellar Fe_3_C that had not yet fully diffused and dissolved during the direct isothermal bainitic transformation for online partitioning, indicating a typical quasi-cleavage fracture. As discussed in Section 3.3, an intergranular crack and transgranular crack (the quasi-cleavage pattern) were clearly observed, as shown in Figure 9d. During the offline tempering, the carbides and second-phase particles did not fully diffuse and dissolve; thus, the change in the impact energy was small. When the offline tempering temperature exceeded 500 °C, numerous carbides and second-phase particles nucleated and grew at the grain boundaries. Simultaneously, the residual austenite decomposed and formed carbides, promoting the accumulation of carbides at the grain boundaries. This led to a strong stress concentration. Therefore, under the action of external impact force, microcracks were formed at the grain boundaries and propagated along the grain boundaries into the grains, inducing a typical cleavage fracture, as shown in Figure 9e. Ultimately, brittle fracture occurred, forming a tempering brittle zone.

## 5. Conclusions

The following conclusions can be drawn from the study.

(1) A new type of low-alloy ultrahigh-strength steel featuring a nanolath structure with excellent mechanical properties was fabricated using the novel Q-P-T process. This process involved direct quenching and isothermal bainitic transformation for partitioning after thermo-mechanical control processing (online Q&P) and offline tempering (reheating and tempering).
(2) The results showed that an ultrafine nanolath martensite/bainite mixed structure, combined with residual austenite in the form of a thin film between the nanolaths, was formed, imparting excellent mechanical properties and toughness at room temperature to the steels.
(3) The various trends observed in the mechanical properties, Brinell hardness, and impact energy at room temperature in the steels during the Q-P-T process can be attributed to the combined effects of the transformation of residual austenite into bainite, the diffusion of C atoms, the formation and dissolution of Fe_3_C and martensite twinning, and the precipitation strengthening of the particles.

## Figures and Tables

**Table 1:** Chemical composition of the material (wt.%).

C	Mn	Si	S	P	Mo	Nb	V	B	Al	Ni	Cr	Fe
0.25	2.2	1.8	≤0.004	≤0.005	0.25	0.06	0.04	0.0025	0.03	1	1.5	Balance

## Data Availability

Data will be made available on request.

## References

[1] Somani M.C., Porter D.A., Karjalainen L.P., Misra D.K. (2013). Evaluation of DQ&P Processing Route for the Development of Ultra-high Strength Tough Ductile Steels. Int. J. Metall. Eng..

[2] Cheng J.H., Lin B.K., Pottore N.S., Sadagopan S., Zhu H., Hu X.H. (2024). A mesoscale crystal plasticity model to predict room-temperature deformation and martensitic transformation of high-strength Quenching and Partitioning (Q&P) Steels and validation with synchrotron X-ray diffraction. Int. J. Plast..

[3] Wang Z.W., Zhang J.F., Xie G.M., Wu L.H., Zhang H., Xue P., Ni D.R., Xiao B.L., Ma Z.Y. (2022). Evolution mechanisms of microstructure and mechanical properties in a friction stir welded ultrahigh-strength quenching and partitioning steel. J. Mater. Sci. Technol..

[4] Pelligra C., Samei J., Shalchi Amirkhiz B., Hector L.G., Wilkinson D.S. (2024). Microstrain partitioning, transformation induced plasticity, and the evolution of damage during deformation of an austenitic-martensitic 1.5 GPa quench and partition steel. Mater. Sci. Eng. A.

[5] Zhang G.F., Shi H.Y., Wang S.T., Tang Y.H., Zhang X.Y., Jing Q., Liu R.P. (2023). Ultrahigh strength and high ductility lightweight steel achieved by dual nanoprecipitate strengthening and dynamic slip refinement. Mater. Lett..

[6] Yuzbekova D., Dudko V., Kniaziuk T., Kaibyshev R. (2024). Tempering behavior of an ultra-high-strength steel with 1.6 wt% Si at low to medium temperatures. Mater. Sci. Eng. A.

[7] Hamada A., Khosravifard A., Ali M., Ghosh S., Jaskari M., Hietala M., Järvenpää A., Newishy M. (2023). Micromechanical analysis and finite element modelling of laser-welded 5-mm-thick dissimilar joints between 316L stainless steel and low-alloyed ultra-high-strength steel. Mater. Sci. Eng. A.

[8] Jovičević-Klug P., Jovičević-Klug M., Thormählen L., McCord J., Rohwerder M., Godec M., Podgornik B. (2023). Austenite reversion suppression with deep cryogenic treatment: A novel pathway towards 3rd generation advanced high-strength steels. Mater. Sci. Eng. A.

[9] Ayer R., Machmeier P.M. (1993). Transmission electron microscopy examination of hardening and toughening phenomena in AerMet 100. Metall. Trans..

[10] Ayer R., Machmeier P.M. (1998). Communications on the characteristics of M2C carbides in the peak hardening regime of AerMet 100 steel. Metall. Mater. Trans..

[11] Lu Y.F., Wang G.L., Zhang M.B., Li R.S., Zhang H.O. (2022). Microstructures, heat treatments and mechanical properties of AerMet100 steel fabricated by hybrid directed energy deposition. Addit. Manuf..

[12] Shi L.Q., Ran X.Z., Zhai Y.M., Pan Y., Zhang S.Q., Cheng X., Tang H.B., Wang H.M. (2023). Influence of isothermal tempering on microstructures and hydrogen-environmentally embrittlement susceptibility of laser additively manufactured ultra-high strength AerMet100 steel. Mat. Sci. Eng. A.

[13] Allen A.J., Gavillet D., Weertman J.R. (1993). SANS and TEM studies of isothermal M2C carbide precipitation in ultrahigh strength AF1410 steels. Acta Metall. Mater..

[14] Li J.H., Zhan D.P., Jiang Z.H., Zhang H.S., Yang Y.K., Zhang Y.P. (2023). Progress on improving strength-toughness of ultra-high strength martensitic steels for aerospace applications: A review. J. Mater. Res. Technol..

[15] Zhang K.Y., Dong W.C., Lu S.P. (2021). Transformation plasticity of AF1410 steel and its influences on the welding residual stress and distortion: Experimental and numerical study. J. Mater. Sci. Eng..

[16] Maloney J.L., Garrison W.M. (1989). Comparison of void nucleation and growth at MnS and Ti2CS inclusions in HY180 steel. Scr. Metall..

[17] Maloney J.L., Garrison W.M. (2005). The effect of sulfide type on the fracture behavior of HY180 steel. Acta Mater..

[18] Gecu R. (2024). Enhancing wear resistance of R220 rail steels by quenching and partitioning (Q&P) treatment. Mater. Lett..

[19] Yin F., Han P.C., Han Q.Y., Wang H.H., Hua L., Cheng G.J. (2024). Ultrastrong gradient M50 bearing steel with lath-shape nano-martensite by ultrasonic shot peening and its enhanced wear resistance at elevated temperature. Mater. Des..

[20] Park J., Jeon J., Seo N., Kang S., Son S.B., Lee S.J., Jung J.G. (2023). Microstructure and mechanical behavior of AISI 4340 steel fabricated via spark plasma sintering and post-heat treatment. Mater. Sci. Eng. A.

[21] Hettig M., Meyer D. (2022). Microstructural influence of consecutive deep rolling of AISI 4140. Procedia CIRP.

[22] Dang J.Q., Zhang H., An Q.L., Lian G.H., Li Y.G., Wang H.W., Chen M. (2021). Surface integrity and wear behavior of 300M steel subjected to ultrasonic surface rolling process. Surf. Coat. Technol..

[23] Wang K., Wen D.X., Li J.J., Zheng Z.Z., Xiong Y.B. (2021). Hot deformation behaviors of low-alloyed ultrahigh strength steel 30CrMnSiNi2A: Microstructure evolution and constitutive modeling. Mater. Today Commun..

[24] Gao Y.K. (2017). Fatigue stress concentration sensitivity and stress ratio effect of a 40CrNi2Si2MoVA steel. Mater. Lett..

[25] Torres-Islas A., Torres-Macias D., Bedolla-Jacuinde A., Guerra F.V., Del-Pozo A., Colin J., Martinez H. (2024). Corrosion behavior and mechanical properties of hot-rolled ultrahigh-strength steel alloys in alkaline and acidic environments. Int. J. Electrochem. Sci..

[26] Speer J., Matlock D.K., De Cooman B.C., Schroth J.G. (2003). Carbon partitioning into austenite after martensite transformation. Acta Mater..

[27] Matlock D.K., Brautigam V.E., Speer J.G. (2003). Application of the quenching and partitioning (Q&P) process to a medium-carbon, high-Si microalloyed bar steel. Mater. Sci. Forum..

[28] Ghosh S., Kaikkonen P., Javaheri V., Kaijalainen A., Miettunen I., Somani M., Kömi J., Pallaspuro S. (2022). Design of tough, ductile direct quenched and partitioned advanced high-strength steel with tailored silicon content. J. Mater. Res. Technol..

[29] Kang S.G., Pierce D., Matlock D.K., Speer J.G., Moor E.D. (2022). Quench and Partitioning Steels. Encycl. Mater. Met. Alloy.

[30] Speer J.G., Moor E.D., Clarke A.J. (2015). Critical Assessment 7: Quench and partitioning. Mater. Sci. Technol..

[31] De A.K., Speer J.G., Matlock D.K. (2003). Color tint-etching for multiphase steels. Adv. Mater. Process..

[32] Speer J.G., Rizzo Assunção F.C., Matlock D.K., Edmonds D.V. (2005). The “quenching and partitioning” process: Background and recent progress. Mater. Res..

[33] Tobata J., Ngo-Huynh K.L., Nakada N., Tsuchiyama T., Takaki S. (2012). Role of Silicon in Quenching and Partitioning Treatment of Low-carbon Martensitic Stainless Steel. ISIJ Int..

[34] Barella S., Gruttadauria A., Menezes J.T.O., Castrodeza E.M., Quaini S.E., Pelligra C., McNally E.A. (2023). The Reliability of Single-Step and Double-Step Quench and Partitioning Heat Treatments on an AISI 420A Low Carbon Martensitic Stainless Steel. Metall. Mater. Trans. A.

[35] Kong H., Chao Q., Cai M.H., Pavlina E.J., Rolfe B., Hodgson P.D., Beladi H. (2017). One-step quenching and partitioning treatment of a commercial low silicon boron steel. Mater. Sci. Eng. A.

[36] Xu P.D., Li C.Y., Li W., Zhu M.Y., Li W., Zhang K. (2022). Effect of microstructure on hydrogen embrittlement susceptibility in quenching-partitioning-tempering steel. Mater. Sci. Eng. A.

[37] Qin S.W., Wang G.G., Zhu Z.M., Song Z.X. (2024). Influence of ultrasonic surface rolling on tensile properties of high carbon low alloy quenching-partitioning-tempering steel. Mater. Sci. Eng. A.

[38] Zurnadzhy V.I., Efremenko V.G., Wu K.M., Azarkhov A.Y., Chabak Y.G., Greshta V.L., Isayev O.B., Pomazkov M.V. (2019). Effects of stress relief tempering on microstructure and tensile/impact behavior of quenched and partitioned commercial spring steel. Mater. Sci. Eng. A.

[39] Zhang J.Z., Zeng L.Y., Zuo X.W., Wan J.F., Rong Y.H., Min N., Lu J., Chen N.L. (2022). Universality of quenching-partitioning-tempering local equilibrium model. J. Mater. Res. Technol..

[40] Speer J.G., Edmonds D.V., Rizzo F.C., Matlock D.K. (2004). Partitioning of carbon from supersaturated plates of ferrite, with application to steel processing and fundamentals of the bainite transformation. Curr. Opin. Solid State Mater. Sci..

[41] Moshtaghi M., Maawad E., Bendo A., Krause A., Keckes J.T.J., Safyari M. (2023). Design of high-strength martensitic steels by novel mixed-metal nanoprecipitates for high toughness and suppressed hydrogen embrittlement. Mater. Des..

[42] Wei F.G., Tsuzaki K. (2012). Gaseous Hydrogen Embrittlement of Materials in Energy Technologies.

[43] Qin W., Szpunar J.A. (2017). A general model for hydrogen trapping at the inclusion- matrix interface and its relation to crack initiation. Philos. Mag..

[44] Safyari M., Moshtaghi M., Hojo T., Akiyama E. (2022). Mechanisms of hydrogen embrittlement in high-strength aluminum alloys containing coherent or incoherent dispersoids. Corros. Sci..

[45] Moshtaghi M., Safyari M. (2023). Different augmentations of absorbed hydrogen under elastic straining in high-pressure gaseous hydrogen environment by as-quenched and as-tempered martensitic steels: Combined experimental and simulation study. Int. J. Hydrogen Energy.

